# Removal of Cr(VI) from Aqueous Environments Using Micelle-Clay Adsorption

**DOI:** 10.1155/2013/942703

**Published:** 2013-10-03

**Authors:** Mohannad Qurie, Mustafa Khamis, Adnan Manassra, Ibrahim Ayyad, Shlomo Nir, Laura Scrano, Sabino A. Bufo, Rafik Karaman

**Affiliations:** ^1^Department of Chemistry and Chemical Technology, Faculty of Science and Technology, Al-Quds University, 20002 Jerusalem, Palestine; ^2^Department of Science, University of Basilicata, Via dell'Ateneo Lucano 10, 85100 Potenza, Italy; ^3^Department of Soil and Water Sciences, The R. H. Smith Faculty of Agriculture, Food and Environment, The Hebrew University of Jerusalem, 76100 Rehovot, Israel; ^4^Department of European Cultures (DICEM), University of Basilicata, Via dell'Ateneo Lucano 10, 85100 Potenza, Italy; ^5^Department of Bioorganic Chemistry, Faculty of Pharmacy, Al-Quds University, 20002 Jerusalem, Palestine

## Abstract

Removal of Cr(VI) from aqueous solutions under different conditions was investigated using either clay (montmorillonite) or micelle-clay complex, the last obtained by adsorbing critical micelle concentration of octadecyltrimethylammonium ions onto montmorillonite. Batch experiments showed the effects of contact time, adsorbent dosage, and pH on the removal efficiency of Cr(VI) from aqueous solutions. Langmuir adsorption isotherm fitted the experimental data giving significant results. Filtration experiments using columns filled with micelle-clay complex mixed with sand were performed to assess Cr(VI) removal efficiency under continuous flow at different pH values. The micelle-clay complex used in this study was capable of removing Cr(VI) from aqueous solutions without any prior acidification of the sample. Results demonstrated that the removal effectiveness reached nearly 100% when using optimal conditions for both batch and continuous flow techniques.

## 1. Introduction

Chromium is a naturally occurring element that exists in rocks, animals, plants, and soil. Although chromium (III) is an essential nutrient that helps our body to use sugar, protein, and fat, chromium (VI) is a toxicant most often used as a pesticide. Pesticides based on Cr(VI) are mainly addressed to preserve wood by commercial formulations containing chromium, copper, and arsenic such as “chromated copper arsenate (CCA)” [1]. Cr(VI) compounds are known to cause cancer in humans who eat or breathe enough of the compound [2]. Chromium compounds can be deposited onto soil and water from airborne particles and there can easily change from one to another oxidation form [3]. Chromium is also discharged into environment during different modern industrial activities, such as electroplating, tanning, plastic surfaces coating for water, and oil resistance [3, 4]. Several treatment methods have been developed to remove chromium from industrial wastewater. They include ion exchange, membrane filtration, reverse osmosis, and adsorption [4–6]. Different adsorbents have been employed to remove chromium from polluted waters such as bentonite [4], activated aluminum and activated charcoal [7], wool [8], soya cake [9], peach kernel and nutshell [10], and functionalized activated carbon [11].

Recently, a new filtration technology for the purification of contaminated water based on either micelle-clay or vesicle-clay complexes has been proposed [12–14]. Both types of organomineral composites include an organic cation and a clay mineral such as montmorillonite (MMT). Octadecyltrimethylammonium (ODTMA) and benzyldimethylhexadecylammonium (BDMHDA) bromides were mostly used for the preparation of micelle-clay complexes [12, 13]. Vesicle-clay complexes were obtained as well using didodecyldimethylammonium (DDAB) as an organic cation [14]. Both types of complexes have a very large surface area and large hydrophobic domains and are typically designed to gain a large excess of positive charge. They can be effective in the removal of water from anionic and neutral pollutants such as herbicides (anionic and hydrophobic ones), anionic detergents, antibiotics and other anionic drugs and components of dissolved organic matter DOM such as fulvic and humic acids [12–15]. 

In this study, the effectiveness of ODTMA-MMT micelle-clay complex for the removal of Cr(VI) anions from water has been tested. The micelle-clay complex used was prepared by mixing MMT with a solution of the cationic surfactant ODTMA-bromide at a concentration higher than its critical micelle concentration (CMC = 0.3 mM) [12]. A filter filled with a mixture of the micelle-clay complex and sand was employed to investigate the ability of the adsorbent to remove Cr(VI) by a continuous flowing technique. The effects of the initial concentration and pH were assessed at constant flow rate and temperature. The feasibility of water filtration to remove Cr(VI) was clearly ascertained.

## 2. Materials and Methods

### 2.1. Chemicals and Instrumentation

All reagents used were AR grade. Stock solutions of Cr(VI) were prepared by dissolving potassium dichromate (K_2_Cr_2_O_7_, Sigma, P5271, USA) in double distilled water. Analytical solutions were prepared by dilution of the stock solution and, when needed, adjusting pH to the desired value by addition of either 1 M HCl or 1 M NaOH. The clay used was Wyoming Na-montmorillonite SWy-2 obtained from the Source Clays Registry (Clay Mineral Society, Columbia, MO, USA). The chemical composition of the montmorillonite is SiO_2_ 62.9%, Al_2_O_3_ 19.6%, Fe_2_O_3_ 3.35%, MgO 3.05%, CaO 1.68%, and Na_2_O 1.53%. The formula of the montmorillonite can be expressed as (Ca_0.12_Na_0.32_K_0.05_)[Al_3.01_Fe(III)_0.41_Mn_0.01_Mg_0.54_Ti_0.02_][Si_7.98_Al_0.02_]O_20_(OH)_4_, as calculated from its chemical composition. The cation exchange capacity (CEC) is 76.4 cmol_+_ kg^−1^. Quartz sand (grain size 0.8–1.5 mm) was purchased from Negev Industrial Minerals (Omar, Israel). ODTMA-bromide and 1,5-diphenylcarbazide were obtained from Sigma Aldrich.

Solution pH was measured using pH-EC-TDS meter, HI 9812, Hanna instruments. Total chromium concentration was determined by Atomic Absorption Spectrometry (AAS) (AA-6200 Shimadzu) [16]. It is worth noting that this method gives total-Cr in solution, irrespective of the oxidation state of the metal. Cr(VI) was determined by UV-vis spectrophotometer (UV-1601 Shimadzu) using the diphenylcarbazide method [17]. 

### 2.2. Micelle-Clay Complex Preparation

The complex was prepared as described previously [12, 18]. Briefly, 10 g L^−1^ MMT was added to 12 mM solution of ODTMA-bromide and stirred for 72 h. Then, the suspension was centrifuged for 20 min at 10,000 g, the supernatant was analyzed for the ODTMA remaining [19] and discarded, and the solid material (micelle-clay complex) was washed with double distilled water and lyophilized. The washing water was analyzed too for ODTMA. The calculated amount of ODTMA adsorbed on MMT was 85%  of the initial concentration.

### 2.3. Batch Experiments and Adsorption Isotherms

100 mL of 20, 50, 100, 150, and 200 mg L^−1^ Cr(VI) solutions was introduced into 250 mL Erlenmeyer flasks. Then, 0.5 g of the micelle-clay complex was added to each Cr(VI) solutions and the obtained suspensions were shaken for 3 h at 25.0 ± 0.2°C. Samples were collected at different times during the adsorption process and analyzed until the equilibrium was reached.

To evaluate the effect of hydrogen ionic concentration on the adsorption reaction, the pH of a series of 50 mg L^−1^ Cr(VI) solutions was adjusted to the desired value in the range from 1 to 8, using either 1 M NaOH or 1 M HCl, and the adsorption process either on pure clay or micelle-clay complex was performed for each of them following the method above.

Equilibrium relationships between adsorbent and adsorbate were obtained by the linear form of the Langmuir adsorption isotherm according to [20]

(1) $$\begin{array}{c} \frac{C_{e}}{Q_{e}}=\frac{1}{kQ_{\max}}+\frac{C_{e}}{Q_{\max}}, \end{array}$$

where *C*_*e*_ is the equilibrium concentration of Cr in mg L^−1^, *Q*_*e*_ is the equilibrium mass of adsorbed chromium in mg per gram of adsorbent, *k* is the Langmuir binding constant (L mg^−1^), and *Q*_max⁡_ is the maximum mass of adsorbed chromium in mg per gram adsorbent.

### 2.4. Column Experiments

Column experiments were performed using glass columns (18 × 4 cm) prepared by mixing 3.0 g of micelle-clay complex and 147 g sand. Sand was thoroughly washed with distilled water and dried at 105 ± 2°C for 24 h prior its use. Wool layer of 2 cm was placed at the bottom of the column to prevent clogging. Cr(VI) solutions of 50 mg L^−1^ having different initial pH in the range of 1–6 were passed through the column at a fixed flow rate of 2 mL min^−1^. 100 mL eluted fractions were collected time after time and analyzed for Cr concentration using AAS. All experiments were conducted in triplicate for statistical analysis.

## 3. Results and Discussion

### 3.1. Batch Experiments

#### 3.1.1. Effect of pH

**Figure 1:**
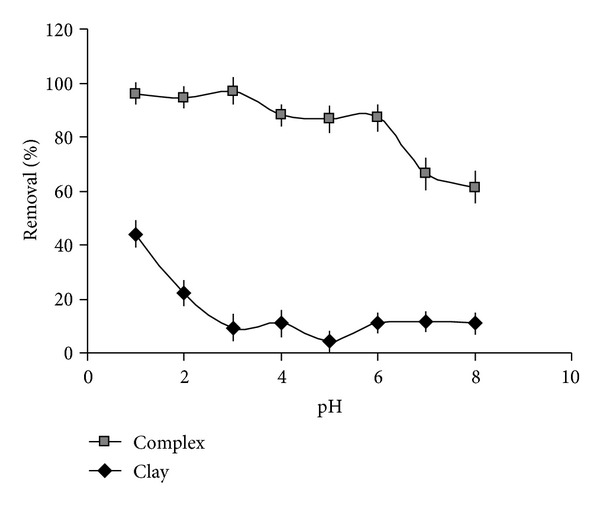
Effect of the initially adjusted pH on the removal of chromium by micelle-clay complex (■) and pure clay (♦). Initial concentration of Cr(VI) = 50 mg L^−1^, contact time = 3 h, temperature = 25.0 ± 0.2°C, and adsorbent dosage = 5.0 g L^−1^. Data represent averages of total-Cr triplicate measurements (AAS) ± SE.

Figure 1 displays the effect of the initially adjusted pH on the removal of total-Cr from water by using either the pure clay or the micelle-clay complex. The maximum adsorption on the micelle-clay complex was obtained at pH between 1.0 and 3.0 (98%). In addition, as striking result it emerged that even at pH = 6 the adsorption was efficient with more that 85% of chromium removal. These results can be explained taking into account that Cr(VI) can give rise to anions with different charge density when exposed to different pH values and undergo transformations into diverse oxidation states.

At pH lower than 6, the predominant ions are possibly HCrO_4_^−^, CrO_4_^2−^, and Cr_2_O_7_^2−^ [20, 23]. These negatively charged ions can be retained electrostatically on the positively charged micelle-clay surfaces. In contrast, the adsorption efficiency for these anions on the negatively charged pure clay is not satisfactory at pH higher than 2. However, at lower pH values, the clay surface can be protonated and consequently its efficiency in removing negative ions can be appreciated reaching 50% of chromium removal [20, 23]. 

**Figure 2:**
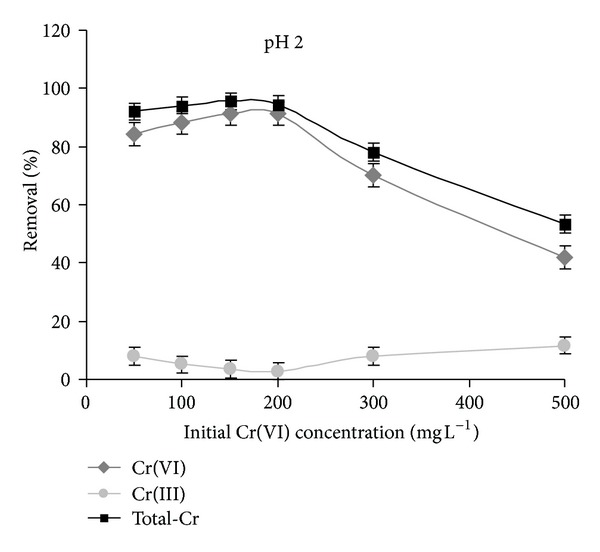
Removal of chromium by micelle-clay complex as total-Cr determined by AAS (■) and as Cr(VI) determined by UV-vis method (♦). Percent of Cr(VI) reduction to Cr(III) (●) is calculated as the difference between total-Cr and Cr(VI) percentages. pH = 2, contact time = 3 h, temperature = 25.0 ± 0.2°C, and adsorbent dosage = 5.0** **g** **L^−1^. Data represent averages of triplicates ± SE.

In order to investigate the possibility of Cr(VI) reduction to Cr(III) upon adsorption onto the complex at different pH values, samples were collected at the end of adsorption trials (equilibrium already reached) performed on purpose at pH 2 and 6, using initial Cr(VI) concentrations 10–500 mg L^−1^, and were analyzed for selective determination of Cr(VI) by UV-vis method [17]. Solutions of Cr(VI) at the same concentrations and pH values used in the adsorption assays were kept in the same environmental conditions without adding the adsorbent and analyzed for Cr(VI) at the end of the time needed to complete the adsorption experiments. As shown in Figure 2, in the initial concentration range 50–200 mg L^−1^, the adsorbed percentages measured as total-Cr and Cr(VI) are very close at pH = 2. However, at initial concentration higher than 200 mg L^−1^, the retention curves of total-Cr and Cr(VI) start to advance farther, suggesting that another oxidation state of Cr can occur in these conditions. Presumably, reduction of Cr(VI) to Cr(III) takes place on the adsorbent followed by the release of Cr(III) into the solution leading to the observed deviation of retention curves. Similar results were also documented in the literature using other adsorbents [21, 22, 24–28]. 

**Figure 3:**
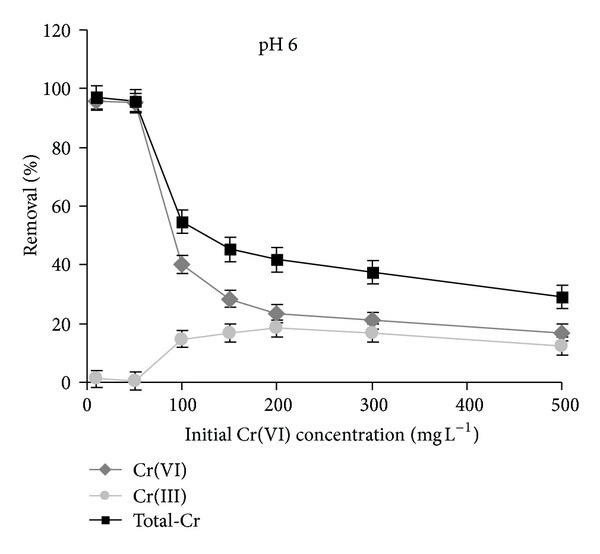
Removal of chromium by micelle-clays complex as total-Cr determined by AAS (■) and as Cr(VI) determined by UV-vis method (♦). Percent of Cr(VI) reduction to Cr(III) (●) is calculated as the difference between total-Cr and Cr(VI) percentages. pH = 6, contact time = 3** **h, temperature = 25.0 ± 0.2°C, and adsorbent dosage = 5.0** **g** **L^−1^. Data represent averages of triplicates ± SE.

Figure 3 shows that at pH 6 the reduction of Cr(VI) occurs at concentration larger than 50 mg L^−1^ and that it is more pronounced than that ascertained at pH 2. On the contrary, in the samples kept without the adsorbent, no appreciable reduction of Cr(VI) to Cr(III) was ascertained at both pH values. The most remarkable observation is that up to pH 6 a total removal of Cr(VI) can be attained at concentration less than 50 mg L^−1^.

#### 3.1.2. Variation of pH during Adsorption

At the end of the adsorption process (equilibrium already reached), the final pH values of the suspensions 50 mg L^−1^ Cr(VI)/5 g L^−1^ of pure clay or micelle clay, in which pH had been adjusted to the desired initial values, were measured and reported in Table 1. For pH values initially adjusted to 1-2, no significant pH variation was observed in both systems. But in the case of initial pH values greater than 2, the final pH increased to subalkaline or alkaline values for both the clay and micelle-clay systems. At very low pH values, the adsorption of Cr(VI) oxoanions on the positively charged surface of micelle-clay system can be mainly electrostatic. Thus, no significant pH changes are observed. On the contrary, at pH values ≥3, the electrostatic adsorption could be not favored, and different specific adsorption mechanisms can take place probably via formation of inner sphere surface complexes. This adsorption process transfers an excess of negative charges, on the surface of adsorbents. In order to compensate the negative charge the latter can adsorb H^+^ from the solution increasing thus the pH. In the case of pure clay, the augment of the pH values may be attributed to (i) the cation exchange capacity of clay surfaces which can adsorb free aqueous protons from the solution and release other cations (mainly Na^+^ and K^+^), (ii) the release of OH^−^ ions, which were previously adsorbed when 1M NaOH had been added to adjust the initial pH value and successively replaced by Cr(VI) oxoanions, and (iii) the adsorption process at higher pH values which can transfer negative charge on the already negatively charged surface of pure clay, inducing the adsorption of hydrogen ions from the solution. These results are in accordance with our previous observations using other low cost adsorbents [6].

#### 3.1.3. Effect of Contact Time

**Figure 4:**
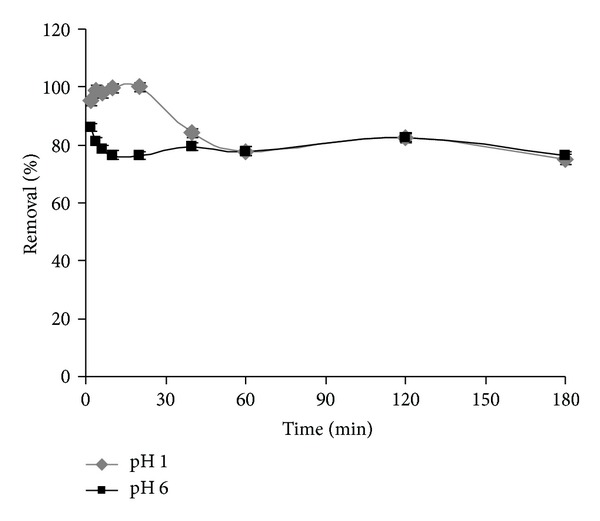
Effect of contact time on the removal of chromium by micelle-clay complex at pH 1 (♦) and pH 6 (■). Initial concentration of Cr(VI) = 50** **mg** **L^−1^, temperature = 25.0 ± 0.2°C, and adsorbent dosage = 5.0** **g** **L^−1^. Data represent averages of total-Cr triplicate measurements (AAS) ± SE.

Figure 4 summarizes the effect of contact time on the percentage of chromium removed by the micelle-clay complex at different initially adjusted pH values, as measured by AAS. In the experiment performed at pH = 1, the removal percentage increased during the first 20 min, and then gradual decrease up to the equilibrium was reached. At pH = 6, the adsorption of Cr(VI) seems initially affected by the competition of hydroxyl ions, but after 60 min the adsorption equilibrium was restored irrespective of pH. It can be speculated that the binding of Cr(VI) to micelle-clays complex was initially regulated by acidity of solution, and then, as discussed above, reduction of Cr(VI) to Cr(III) can take place at a later stage, and chromium remains in the solution as unadsorbed fraction due to the positive charge of Cr(III). Similar results have been also reported in the literature using other low cost adsorbents [21, 22, 24–28]. 

#### 3.1.4. Adsorption Isotherm

**Figure 5:**
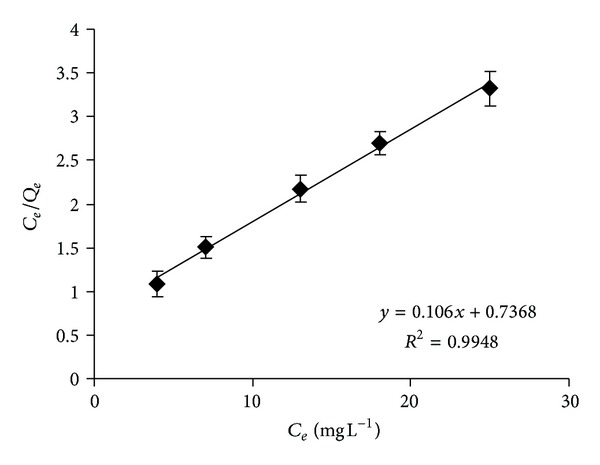
Langmuir isotherm plot for the adsorption of chromium into the micelle-clay complex. Contact time = 3** **h, temperature = 25.0 ± 0.2°C, and adsorbent dosage = 5.0** **g** **L^−1^. Data represent averages of total-Cr triplicate measurements (AAS) ± SE.

Langmuir isotherm is the most widely used method for modeling the equilibrium data and for the determination of the adsorption capacity. The Langmuir equation (1) was applied to quantify adsorption capacity of Cr(VI) by micelle-clays complex measured as total-Cr by AAS. The adsorption process performed at pH = 6 fitted a linear plot only in the low equilibrium concentration region as shown in Figure 5. The Langmuir constants *Q*_max⁡_ and *k* were calculated from the slope and the intercept of (1) giving values of 9.43 mg g^−1^ and 0.144 L mg^−1^, respectively. In comparison, the adsorption of the monovalent anionic drug diclofenac by the micelle-clay composite [15] was characterized by 16-fold larger *Q*_max⁡_ and by 2-fold smaller *k*.

Comparing our results for the adsorption parameters (*Q*_max⁡_ and % removal) at pH = 6 with those reported in the literature at the same pH (Table 2), we observe that the removal percentage obtained using different low cost adsorbents was in the range of 25–57%, in which values are significantly lower than 86% achieved in this study. This finding supports the possibility of using the micelle-clay complex as an efficient adsorbent for the removal of Cr(VI) from water at ambient pH without any significant pH adjustment.

**Figure 6:**
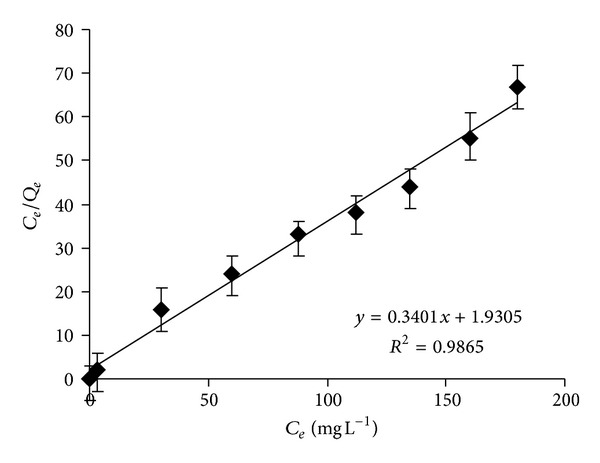
Langmuir isotherm plot for the adsorption of Cr(VI) into the micelle-clay complex. Contact time = 3** **h, temperature = 25.0 ± 0.2°C, and adsorbent dosage = 5.0** **g** **L^−1^. Data represent averages of Cr(VI) triplicate measurements (UV-vis) ± SE.

In order to selectively investigate the adsorption isotherm for Cr(VI), we analyzed the equilibrium concentration (*C*_*e*_) of Cr(VI) using the UV-vis method.  Figure 6 displays the Langmuir isotherm plotted using new *C*_*e*_ data. In this case, the linearity of the isotherm was maintained within the full range of equilibrium concentrations measured. The Langmuir constants *Q*_max⁡_ and *k* were calculated from the new slope and the intercept of (1) obtaining values of 2.84 mg g^−1^ and 0.176 L mg^−1^, respectively. The new value of *Q*_max⁡_ was lower than that obtained determining *C*_*e*_ as total-Cr values. This result confirms that micelle-clay system was catalyzing the reduction of adsorbed Cr(VI) to Cr(III) and then the release of the last species to solution. In any case, micelle clay complex showed the highest maximum adsorption capacity compared to other low cost adsorbents.

### 3.2. Column Experiments

**Figure 7:**
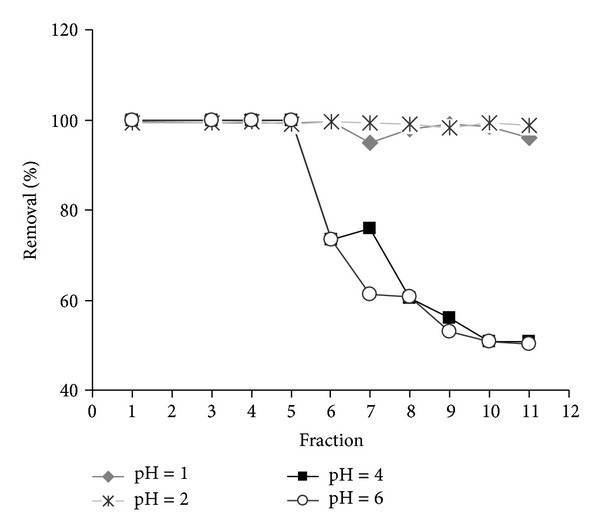
Removal percentage of chromium at pH = 1, 2, 4, and 6 using column filtration. Flow rate = 2** **mL min^−1^, eluted Cr(VI) concentration = 50** **mg** **L^−1^, and temperature = 25.0 ± 0.2°C. Volume of each collected fraction = 100** **mL. Data represent averages of triplicates (standard errors have been omitted to avoid crowding of symbols).

Figure 7 displays the breakthrough curves representing the removal efficiency of Cr(VI) from solutions at different pH values by column filtration on micelle-clay complex/sand system as described in Section 2. Results indicate that complete removal of chromium was achieved at all studied pH values. However, at pH 1 and 2, the breakthrough point was greater than 1000 mL, whereas at pH 3, 4, and 6, the saturation point was significantly lower with a value of about 500 mL. These results are consistent with those obtained from batch experiments, indicating that the elution volume plays an important role during the adsorption process at pH values higher than 2. Column experiments showed complete removal of Cr(VI) with possible reduction to Cr(III) after the breakthrough points.

In a recent paper [29], dealing with the removal of Cr(VI) ions from aqueous solutions by continuous adsorption on ethylamine modified chitosan carbonized rice husk composite beads (EAM-CCRCBs), the best removal conditions for Cr(VI) ions were previously determined using batch experiments, obtaining optimized parameters such as adsorbent dosage (0.14 g), chromium salt initial concentration (300 mg L^−1^), and pH value (pH 2). In the column adsorption experiments, authors used two salt concentrations (100 and 300 mg L^−1^) and different column height, observing that the lower the bed heights the faster the breakthrough curve could be obtained, and either with the higher concentration or higher flow rate, the adsorbing bed was saturated in shorter time.

## 4. Conclusion

It can be concluded that the micelle-clay complex prepared by mixing montmorillonite with ODTMA bromide is an efficient adsorbent for Cr(VI) removal from aqueous medium at ambient pH since this material showed a relatively large adsorption capacity, which renders it more efficient in removing Cr(VI) from water environments than other low cost materials.

The regeneration of the adsorbent from Cr as well as the mechanism of its removal is currently being investigated.

## Figures and Tables

**Table 1:** Initial and final pH during adsorption of Cr(VI) either on pure clay or micelle-clay complex. Initial concentration of Cr(VI) = 50 mg L^−1^, contact time = 3 h, temperature = 25.0 ± 0.2°C, and adsorbent dosage = 5.0 g L^−1^. Data represent averages of triplicates.

Initial pH	Final pH	
	Clay	Micelle-clay complex
1.00	0.99 ± 0.03	1.05 ± 0.04
2.00	2.01 ± 0.03	2.00 ± 0.04
3.00	6.52 ± 0.04	6.04 ± 0.04
4.00	7.00 ± 0.04	6.71 ± 0.04
5.00	7.17 ± 0.05	6.98 ± 0.05
6.00	7.67 ± 0.05	7.36 ± 0.04
7.00	7.75 ± 0.05	8.17 ± 0.03

**Table 2:** Comparison between the efficiencies of different low cost adsorbents towards removal of Cr(VI) at pH = 6 and temperature = 25.0 ± 0.2°C.

Adsorbents		Adsorption capacity of Cr(VI) mg g^−1^	Removal%
(1)	Bagasse [21]	0.0005	30
(2)	Fly ash [21]	0.001	25
(3)	As received CSC [22]	2.2	55
(4)	CSC coated with chitosan [22]	3.65	57
(5)	Micelle-clay complex (this work)	9.43	86
